# Ethnopharmacology, Phytochemistry, and Pharmacology of *Syzygium nervosum*

**DOI:** 10.1155/2020/8263670

**Published:** 2020-11-02

**Authors:** Giang Nam Pham, Tu Thanh Thi Nguyen, Hieu Nguyen-Ngoc

**Affiliations:** ^1^Division of Biotechnology, Vietnam-Korea Institute of Science and Technology, Hanoi 10000, Vietnam; ^2^Faculty of Traditional Medicine, Hanoi Medical University, Hanoi 10000, Vietnam; ^3^Faculty of Pharmacy, PHENIKAA University, Hanoi 12116, Vietnam; ^4^PHENIKAA Research and Technology Institute (PRATI), A&A Green Phoenix Group JSC, No. 167 Hoang Ngan, Trung Hoa, Cau Giay, Hanoi 11313, Vietnam

## Abstract

*Syzygium nervosum*, which belongs to the Myrtaceae plant family, is widely distributed and cultivated in South East Asian countries. The decoction of *S. nervosum* leaves and flower buds has been consumed regularly as a beverage among the Vietnamese and Chinese communities. In addition, it has also been used in traditional medicine for a variety of purposes, notably for influenza, skin diseases, and digestive conditions. To date, there has been a considerable number of publications on chemical profiling and pharmacological activities of *S. nervosum* crude extract and pure isolated compounds. Our analysis indicated the characteristic chemical scaffolds and potential bioactivities on cancer, diabetes, and inflammatory diseases of this plant. The review aims to summarize up-to-date past study results and suggest future research direction on this species, in order to promote clinical applications of *S. nervosum*.

## 1. Introduction

*Syzygium nervosum* (synonyms: *Cleistocalyx nervosum*, *Cleistocalyx operculatus*, and *Eugenia operculata*; common names: “Voi” in Vietnamese, “Shui weng” in Chinese) is widely distributed in tropical areas of South East Asian countries [1–3]. Hot water brewing of *S. nervosum* leaves or flower buds has been commonly consumed as a beverage in Vietnam and China, and it is well known for treating influenza and some digestive conditions according to traditional medicine [1–5]. Besides that, *S. nervosum* leaves and flower buds were also used externally for inflammatory conditions, including bruises, acnes, and skin ulcers [2, 5, 6].

In the period of 2002–2019, phytochemical studies have identified a total of 86 natural compounds from leaves and flower buds of *S. nervosum*. The main components of *S. nervosum* were determined as oleanane-and ursane-type triterpenoids [7], C-methylated flavonoids [8], and polycyclic phloroglucinols [9, 10]. Of these, C-methylated chalcones were considered as major and pharmacologically responsible constituents of this medicinal plant. *S. nervosum* crude extract and its major constituent, 2′, 4′-dihydroxy-6′-methoxy-3′, 5′-dimethylchalcone (or DMC), were also examined in a variety of pharmacological assays. To a certain extent, some pharmacological effects explained the medicinal uses of this plant in folklore medicine, such as antiviral [8, 11], anti-inflammatory [12], and antioxidant activities [13, 14]. Furthermore, other interesting bioactivities were also discovered, such as anticancer [15–19] and antidiabetic [20, 21] properties, which greatly contributed to the potential clinical application of this plant.

In this paper, a comprehensive review related to ethnopharmacology, chemical constituents, and pharmacological studies of *S. nervosum* and its isolated compounds was presented. The authors expect that this review could provide an overview of the current status of *S. nervosum* research and a constructive perspective for upcoming studies, in order to encourage the development of clinical therapeutics using this plant in the near future.

## 2. Ethnobotany and Ethnopharmacology

*S. nervosum* is a branched tree that can be up to 15 m high with thick brown-to-black bark. The leaf has an elliptic shape with 8–20 cm long × 5–10 cm wide. Both leaf surfaces have glandular punctate with many secondary veins. Flower buds are oval with a size of 4–6 mm long × 2-3 mm wide and have bell-shaped gray calyx. Flowers are pale green when fresh and grayish when dried. Fruits have spherical to broadly ovoid shape with violet to black color when mature. Leaves, young branches, and flower buds have a characteristic pleasant fragrance [1, 3, 4].

The documentation in traditional Vietnamese medicine has recorded that *S. nervosum* leaves and flower buds were used to treat digestive conditions, abdominal pain, and diarrhea. Besides that, the decoction of flower buds was externally used for the treatment of wounds, itchy sores, and acne while the barks were used for antiseptic effects [1, 3, 4]. In Chinese folk medicine, *S. nervosum* leaves and barks were used externally to treat skin ulcers, scabies, and other skin diseases; when used internally, the leave extraction was for the treatment of diarrhea, pimples, and breast inflammation [2, 6]. The water extraction of flowers was also used for the treatment of influenza, dysentery, and indigestion [5] while the roots were used for jaundice and abdominal pain [2].

## 3. Phytochemistry

Phytochemical studies on *S. nervosum* flower buds and leaves resulted in the isolation and identification of a total of 86 compounds, which can be categorized into three main groups, including terpenoids, flavonoids, and phloroglucinols. The structures were elucidated by standard spectroscopic methods (1D and 2D NMR, MS). The absolute configuration of complex structures was determined by signs of cotton effect in circular dichroism spectrum [11], single-crystal X-ray diffraction [9, 10], or quantum chemical calculations [9, 10]. All compound names and respective references are summarized in Table 1.

### 3.1. Terpenoids

Isolated terpenoids from *S. nervosum* are mostly oleanane- (**6**–**9**, **26**–**33**) and ursane-type (**1**–**5**, **10**–**20**) triterpenoids. Oleanane-type triterpenoid is also known as *β*-amyrin triterpenoid, which consists of five six-membered rings (A-E) with *trans*-conformation of A/B, B/C, and C/*D* rings and *cis*-conformation of D/E rings. Ursane-type triterpenoid (*α*-amyrin type) is nearly identical to oleanane-type from a chemical perspective, except for two methyls at C-19 and C-20 (in oleanane-type, both methyls connect to C-20).

**Figure 1:**
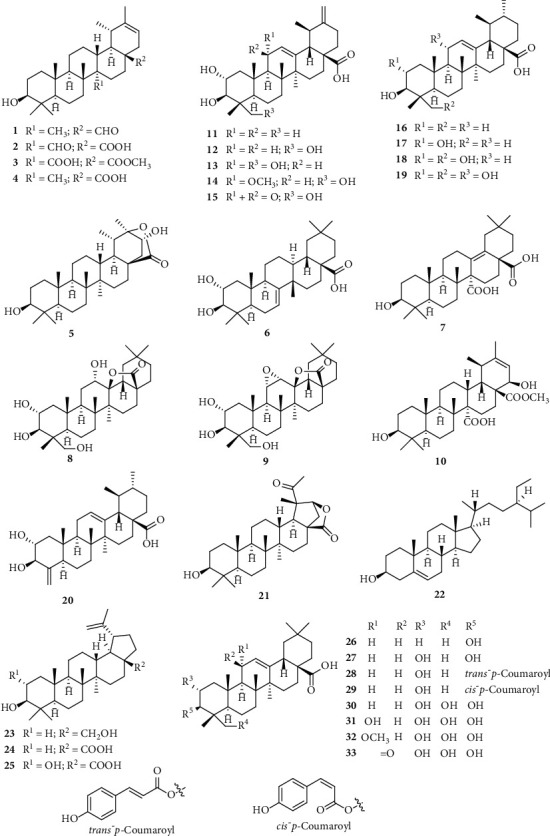
Isolated terpenoids from *S. nervosum*.

Naturally occurring triterpenoids from *S. nervosum* usually possess a carboxylic group at the C-28 position, along with the additional hydroxy or carboxylic or epoxy at various positions. Noticeably, there have been several compounds, in which an intramolecular ester (lactone) functionality can be observed (**5**, **8**, **9**, and **21**). Besides that, three lupane-type triterpenoids (**23**–**25**) and two unusual megastigmane derivatives (**73** and **74**) were also isolated and structurally elucidated (Figure 1).

### 3.2. Flavonoids

**Figure 2:**
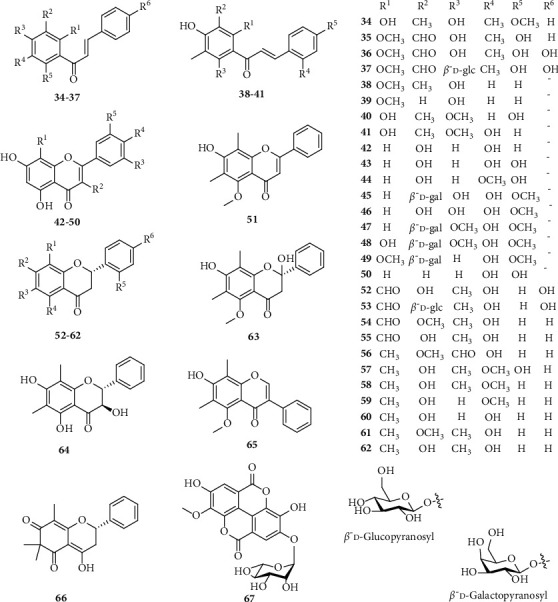
Phenolic compounds from *S. nervosum*.

C-methylated flavonoid is the characteristic group of the compound in *S. nervosum* with the dominant occurrence of DMC (**38**). Unlike common flavonoids, C-methylated derivatives possess one or two methyl groups directly linked with the aromatic ring (**34**–**41**, **51**–**65**). Besides that, ubiquitous flavonoids and their glycosides were also identified, such as kaempferol (**42**), quercetin (**43**), tamarixetin (**44**), myricetin derivatives, and their glycosides (**45**–**47**) (Figure 2).

### 3.3. Phloroglucinols

**Figure 3:**
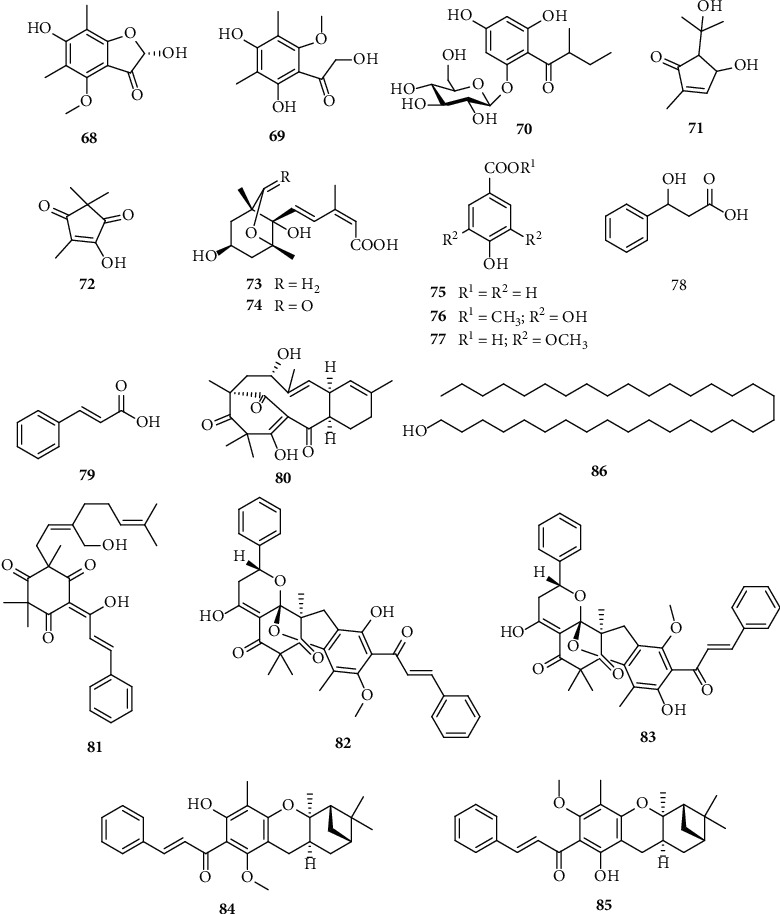
Phloroglucinols and miscellaneous compounds from *S. nervosum*.

“Phloroglucinol” refers to a group of compounds that are biosynthesized via the condensation of three malonyl-CoAs, followed by the cyclization of 3,5-diketoheptanedioate. Naturally occurring phloroglucinols usually possess an acyl functionality along with three hydroxyl groups. The phloroglucinols isolated from *S. nervosum* possess characteristic structural features, such as C-linked methyl groups in the aromatic ring (**66**, **68,** and **69**), the hybridization of terpene and acylphloroglucinol moieties (**80**, **81**, **84**, and **85**), and polycyclic phloroglucinol dimers (**82** and **83**) (Figure 3).

## 4. Analytical Studies

There have been several published analytical studies for the quantification of major compounds from *S. nervosum*. The high-performance liquid chromatography (HPLC) was chosen for its relatively short analytical time, baseline peak separation, and applicability for many compound groups. Phytochemical investigations on *S. nervosum* flower buds revealed that flavonoids and triterpenoids are major bioactive compounds. Ye et al. [28] developed an HPLC method to quantify DMC (**38**) and ursolic acid (**16**). The analytical procedure was conducted on a ZORBAX Eclipse XDB-C_18_ (5 *μ*m, 4.6 mm × 250 mm), a C_18_ reverse-phase column, eluting with an isocratic program (methanol (A); 0.2% aqueous H_3_PO_4_ (B); A : B = 93 : 7, v/v), UV wavelength at 220 nm, and analytical time of approximately 10 min. The validated method was then applied to quantify the content of two compounds in three samples collected from different locations. As a result, the contents of DMC (**38**) and ursolic acid (**16**) were determined in the range of 0.973–1.201 mg/g and 2.857–3.549 mg/g of dried plant materials, respectively [28].

DMC (**38**) was chosen as the analytical marker of *S. nervosum* for its high content and potential bioactivity. The quantification method was developed and validated on C18 reverse-phase HPLC column, mobile phase consisting of organic solvent (acetonitrile or methanol) and aqueous solvent (containing phosphoric acid or trifluoroacetic acid), and UV detection set at ∼330 nm, which is a maxima UV wavelength of DMC (**38**). These quantification studies revealed the content of DMC in flower buds ranging from 0.75% to 1.85% in different extracts [12, 29].

Tran et al. [24] quantified the content of maslinic acid (**27**) and its coumaronyl derivatives (**28** and **29**), which were potent RANKL-induced osteoclast formation inhibitors, by HPLC. The optimal condition was performed on a Sunfire C_18_ column (4.6 × 150 mm, 5 *μ*m), eluting with gradient of acetonitrile and 0.1% trifluoroacetic acid in the water, and UV lamp set at 205 nm. The content of compounds **27**–**29** was determined as 7.20, 0.16, and 0.52%, respectively [24].

Besides that, the total phenolic content of *S. nervosum* was also quantified to link with pharmacological effects. The common method is using the Folin–Ciocalteu method. In this method, the extract is diluted and shaken with Folin–Ciocalteu reagent (a mixture of phosphomolybdate and phosphotungstate). After that, the absorbance of the mixture was measured by a spectrophotometer at specific UV wavelengths (commonly at ∼500 and ∼750 nm). Gallic acid and catechin were usually chosen as reference standards, and the total phenolic content was expressed as gallic acid (GAE) or catechin equivalents (CE). The total phenolic content of *S. nervosum* was determined in different plant parts as follows: flower bud (58.5–157.7 mg GAE/g [30, 31]; 122.5 mg CE/g [20]), leaves (1.03 mg–511.4 mg GAE/g) [30, 32], and berry (5.35–61.1 mg GAE/g) [33, 34].

Noticeably, Zhang and Lu [31] compared the total polyphenol and DMC (**38**) content obtained from different extraction solvents (0, 30, 60, 95% ethanol, petroleum ether, and ethyl acetate extracts). As a result, the total polyphenol content ranked in the order of “aqueous extract > 30% ethanol extract > 60% ethanol extract > 95% ethanol extract > petroleum ether extract > ethyl acetate extract.” The petroleum ether extract contained the highest content of DMC (1.85%), followed by ethyl acetate extract (1.14%), 95% ethanol extract (0.75%), 60% ethanol extract (0.59%), 30% ethanol extract (0.23%), and aqueous extract (not detected). The authors also pointed out the close relation between pancreatic *α*-amylase inhibitory effects and total polyphenol content; however, the content of DMC (**38**) was determined to be a decisive factor for inhibitory activity on pancreatic lipase [31].

## 5. Synthesis of Bioactive Compounds from *S. nervosum* and Their Analogues

Since DMC (**38**) is an interesting bioactive compound with a wide range of pharmacological effects, the efforts of DMC mass production are necessary for further research and clinical application. Although DMC was determined as the major constituent of *S. nervosum* flower buds, its content was not high enough for industry-scaled extraction and isolation. Mass production of bioactive compounds by microbial fermentation has recently become a new approach to deal with this question of research. Wang et al. [35] published a study on optimizing conditions for biosynthesizing DMC from *Ceriporia lacerate* DMC11-6, a new endophytic fungus. From an optimal fermentation medium, DMC can be produced in a high amount (100.24 ± 1.60 *μ*g/L). The authors also suggested that further investigation using genetic tools to overexpress DMC-biosynthesizing genes is required [35].

Compound **35** was first isolated and structurally elucidated by Ye et al. [36] with moderate anticancer activities. In order to shed light on the structure-activity relationship of bioactive chalcones from *S. nervosum*, Zhuo et al. [37] proposed a concise synthesis of 3′-formyl-4′,6′-dihydroxy-2′-methoxy-5′-methylchalcone (**35**) and its series of B-ring-modified analogues from 2,4,6-trihydroxyacetophenone. The results showed that the analogue with bromide substitution at C-4 on B-ring exhibited a significant improved antiproliferative activity with IC_50_ values ranging from 10.8 ± 0.9 to 20.2 ± 1.6 *μ*M, which was 10 times more potent compared to the lead compound (**35**) [37]. In another synthetic study, Wang's group dealt with the hydrophobic nature of DMC, which made the compound barely soluble in water, leading to the limited application for future clinical use. The authors synthesized a series of DMC analogues with different ionizable groups, which improved significantly hydrophilic properties of the DMC scaffold, as well as anticancer activities against various types of cancer cells [38].

Although there has been a modest number of synthetic publications on bioactive compounds from *S. nervosum*, the natural-product-inspired synthetic approach should be taken into consideration for the future development of novel chemical entities for targeted diseases. However, the past studies only focused on C-methylated flavonoids and their analogues but neglected other bioactive compound groups, which are also found from the plant in considerable amounts, for example, triterpenoids and phloroglucinols. Future studies should pay more attention to that area of research.

## 6. Pharmacological Activities

### 6.1. Anticancer Activities

#### 6.1.1. Cytotoxicity against Different Cancer Cell Lines

There have been studies on cytotoxicity of DMC (**38**), the major chalcone isolated from *S. nervosum*, on different cancer cell lines. The half-maximal inhibitory concentration (IC_50_) and respective cancer cell lines are summarized in Table 2.

In general, DMC was found to be active against many cancer cell lines with different magnitude of cytotoxicity. The chalcone exhibited the strongest cytotoxicity against A549 cells with an IC_50_ value of 2.3 ± 0.44 *μ*M. DMC was also very active against a variety of cancer cell lines with IC_50_ ranging from 8 to 15 *μ*M, such as HepG2, ASK, P-388, and PANC-1. For other cancer cell lines, DMC only showed moderate activities (IC_50_ 30–85 *μ*M).

However, there does exist inconsistency among reported data on cytotoxicity of DMC in the same cancer cells from independent pharmacological studies [25, 38]. The variation in IC_50_ may be due to the differences in the evaluation methods. Currently, the most common method for cytotoxicity measurement is the MTT assay, which was applied to identify the IC_50_ of DMC in a study by Wang et al. [38, 40]. However, Chailungka et al. used the TCA method, in which treated cells were trypsinized and resettled in the new culture plates before the TCA measurement [25]. Consequently, a more complicated process may result in differences in IC_50_ identification. Furthermore, the concentration of DMSO is also an issue. Since DMSO was reported to expose cytotoxicity in a dose-dependent manner in certain cell lines [41], therefore, the differences in DMSO concentration used as a negative control in different measurement methods may also cause the variety of IC_50_ values of DMC in the cytotoxicity assay.

#### 6.1.2. Reversal Effects of DMC (**38**) and Its Analogues on Drug-Resistant Cancer Cell Lines

Multidrug resistance is one of the major challenges for the chemotherapeutic treatment of cancer. Noticeably, cotreatment of DMC (**38**) with anticancer drugs, such as 5-fluorouracil (5-FU), Taxol, and doxorubicin against drug-resistant cancer cells resulted in enhanced drug sensitivity in cancer cells. Specifically, Qian et al. [15] investigated the reversal effect of DMC against doxorubicin-resistant KB-A1 cells. In the presence of DMC, the IC_50_ value of doxorubicin against KB-A1 cells dropped from 13.9 ± 0.7 to 3.6 ± 0.7 *μ*g/mL. In the xenograft model, DMC (20 and 40 mg/kg) potentiated the anticancer effects of doxorubicin, which was evidenced by 47.0 and 70.0% reduction of the tumor weight, respectively. The underlying mechanism was through the attenuation of MDR1 gene expression and the reduction of intracellular P-glycoprotein [15].

Cotreatment of DMC (4 and 8 *μ*M) enhanced the chemosensitivity of BEL-7402/5-FU cells to 5-FU by 3.68 and 4.92 times, respectively, while the figures for doxorubicin were 1.71- and 2.35-fold. Flow cytometry also showed the increased apoptotic cell population when using low-dose DMC with 5-FU, compared to DMC or 5-FU sole treatment [17]. A follow-up study from the same group reported the reversal effects of DMC in drug-resistant cancer cells in the human hepatocellular tumor xenograft model. A combination of low-dose DMC and 5-FU significantly inhibited the growth of BEL-7402/5-FU cell xenografts (55.6% reduction of tumor weight), compared to the inhibition rate of 22.2% and 16.7% when using 5-FU and DMC alone, respectively. In addition, the cotreatment of DMC (20 and 40 mg/kg) also increased the accumulation of 5-FU inside tumor tissue and significantly elevated the activity of caspase-3, which plays a central role in the execution phase of cell apoptosis [16]. The underlying mechanism of DMC against the multidrug-resistant BEL-7402/5-FU hepatocellular carcinoma cells was determined to be through the mitochondria-dependent apoptotic pathway and increasing the generation of ROS inside the cells. Moreover, DMC was found to block cell cycle progression at the G1 phase by downregulating the levels of related proteins, including p-GSK3*β*, cyclin D1, and CDK4 [42].

To develop new anticancer agents from the DMC backbone, Wang et al. [35] synthesized a series of water-soluble DMC analogues with ionizable tertiary amine groups. These synthetic compounds were then tested alone or in a combination with Taxol. Consequently, all DMC synthetic analogues showed strong antiproliferative activities on different cancer cell lines (IC_50_ values from 1.3 ± 0.16 to 24.7 ± 3.94 *μ*M), and they also displayed synergistic effects with Taxol against a multidrug-resistant cancer cell line (Hela/Tax). The most promising synergistic effects were quantified by the combination index (CI < 0.15) and dose-reduction index (DRI > 15) [38].

To summarize, the cotreatment of DMC with other well-known anticancer drugs, such as 5-FU, doxorubicin, or Taxol, was shown to enhance the activities of the drugs, increase the accumulation of drugs in tumor tissues, and also even have synergistic effects with drugs when treated in resistant cancer cells. Further studies, especially in animal models, should be done to clarify the application of DMC in cancer treatment.

#### 6.1.3. Chemopreventive Effects

Treatment of aqueous extract of *S. nervosum* fruit (1000 mg/kg) significantly reduced the number and size of glutathione-S-transferase placental form (GST-P)-positive foci induced by diethylnitrosamine (a rodent genotoxic compound) and phenobarbital (hepatocarcinogenic promoter), which is an important indicator for early stages of rat liver carcinogenesis. Moreover, the extract also increased remarkably the enzymatic activities of hepatic glutathione peroxidase and catalase, in comparison with the carcinogen-treated group; however, the extract did not affect the activities of glutathione reductase and heme oxygenase in rat liver. The study also suggested that the chemopreventive effects of *S. nervosum* might be due to anthocyanin constituents, which are antioxidant enhancers [18].

In another study, Inboot et al. [43] set up a *Salmonella* mutation assay to evaluate the antigenotoxic effects of *S. nervosum* seed extract. The results showed that the seed methanol extract was capable of inhibiting aflatoxin B1, MeIQ (2-amino-3,4 dimethylimidazo [4,5-*f*]quinolone)-induced mutagenesis dose-dependently, which indicated that the mode of action of *S. nervosum* seed extract might be through inhibitory effects on carcinogen-bioactivated enzymes rather than direct action on mutagens [43].

Studies on preventive effects of *S. nervosum* against carcinogens are still in the early stage, which still requires more works to draw any conclusion for the preventive benefits of the plant against cancer.

#### 6.1.4. Underlying Mechanism of Anticancer Activities of DMC

The molecular mechanisms of antitumor effects of DMC (**38**) on different models were also investigated. In an *in vivo* study on a solid human tumor xenograft (SMMC-7721) mouse model, DMC with a dose of 150 mg/kg was shown to significantly reduce the average tumor weight in 50 days (DMC group: 0.59 ± 0.12 g; control: 1.42 ± 0.11 g). The study also identified that the lethal dose of DMC was 3800 mg/kg, which can be considered only slightly toxic [19]. The cytotoxic potential of DMC on human hepatoma cells (SMMC-7721) was determined via inhibiting intracellular ROS level and inducing the loss of mitochondrial membrane potential, increasing caspase-3 and caspase-9 activities, and downregulating the expression of Bcl-2 protein. Interestingly, DMC shows almost no toxicity against human normal liver (L-02) and human normal fetal lung fibroblast (HFL-1) cells [44, 45]. Other studies of anticancer effects of DMC on different cancer cell lines (K562 (leukemia cells) or PANC-1 (pancreatic cancer cells)) also indicated that the chalcone could lower Bcl-2/Bax ratio by decreasing the expression of Bcl-2 gene and activating caspase-3 and caspase-9 [39, 46].

The past study results indicated that the DMC exhibited anticancer activities via multiple mechanisms of action, including genetic expression of apoptotic proteins (Bcl-2 and Bax), enhancing caspase-3 and caspase-9 activities, or ROS scavenging. Further works should be done to understand the biological mechanism of DMC in both cellular and animal setups.

### 6.2. Antidiabetic and Antiobesity Activities

There have been a number of studies on antidiabetic and antiobesity effects of *S. nervosum* extracts and the major compound (DMC, **38**). The extracts of *S. nervosum* were shown to inhibit the activities of various digestive enzymes *in vitro*, including *α*-glucosidase, maltase, sucrase [20], lipase, and *α*-amylase [31]. It was also found out that the inhibitory effects of *S. nervosum* were relevant to total polyphenol content, particularly DMC [31]. *In vivo*, *S. nervosum* aqueous extract (2 g/kg) also reduced postprandial glucose level of STZ-induced diabetic rats after oral loading of 2 g/kg of maltose, which was comparable to that of acarbose (25 mg/kg). The extract (500 mg/kg) was also shown to reduce blood sugar levels in fasting rats with maximal reduction after 6 hours. The authors also studied the long-term hypoglycemic effects of *S. nervosum* by giving the extract (500 mg/kg) orally to diabetic rats in 8 weeks. As a result, the *S. nervosum* extract remarkably reduced the blood glucose level and urine volume, and no sign of toxicity was observed during the study [20]. Another study performed by the same group also indicated that the aqueous extract of *S. nervosum* (500 mg/kg) showed protective activities on *β*-cells of pancreatic islets in the STZ-induced diabetic rat model after 9 weeks of treatment. Histopathological results showed that there were less severe degenerative and necrotic changes in the pancreatic islets and immunohistochemical staining displayed the large area of strong insulin antigen reactivity in *β*-cells in the islets of Langerhans in the *S. nervosum*-treated group [21].

2′,4′-dihydroxy-6′-methoxy-3′,5′-dimethylchalcone, or DMC (**38**), the major compound of *S. nervosum* flower buds, has also become a compound of interest for its potent antidiabetic and antiobesity activities via multiple mechanisms of action. DMC exhibited strong noncompetitive inhibitory effects on pancreatic *α*-amylase (IC_50_ 43 *μ*M) but literally ineffective against intestinal *α*-glucosidase (∼20% inhibition at 200 *μ*M). In addition, DMC was also capable of inhibiting glucose transporter 2 (GLUT 2; 54% inhibition at 150 *μ*M), which subsequently blocked the reabsorption of glucose in the kidney and therefore indirectly lowered the glycemic level. The authors also tested the protective effects of DMC against H_2_O_2_-induced insulin reduced secretion in MIN6 cells. As a result, the cellular viability of MIN6 cells with DMC pretreatment showed no significant changes when compared with control [47]. In another study, Hu et al. [48] studied the insulinotropic effects of DMC on glucotoxicity in RIN-5F *β*-cells. As a result, DMC cotreatment could stimulate insulin secretion from *β*-cells dose-dependently without affecting the physiological insulin secretion. The underlying mechanism was determined through the upregulation of the GLP-1 receptor and the activation of PDX-1, PRE-INS, and GLUT2-GCK signaling pathway. Besides that, DMC also reduced NO production and MCP-1 expression, which indicated the protective effects of DMC against cellular damage and oxidative stress in pancreatic islets [49]. DMC (10 *μ*M) also increased glucose uptake in differentiated adipocytes (3T3-L1). Noticeably, at high concentration (10 and 20 *μ*M), DMC remarkably suppressed the lipid accumulation in 3T3-L1 cells while at low concentration (2.5 *μ*M), DMC stimulated the lipid storage in the same experimental cell line. The authors hypothesized that DMC upregulated the expression PPAR-*γ* at a high dose and suppressed at low-dose [48]. Luo and Lu [50] also studied the protective effects of DMC on H_2_O_2_-induced cell damage in MIN6 (mouse insulinoma) cells. Pretreatment of DMC (12.5 and 25 *μ*M) was shown to improve mitochondrial functions by reducing nucleus fragmentation, decreasing the intracellular level of ROS, and preventing the loss of mitochondrial potential [50].

Choi et al. [51] investigated the antidiabetic effects of DMC (**38**) in both *in vitro* and *in vivo* models. DMC was shown to increase glucose uptake and promote fatty acid oxidation (FAO) via AMPK activation in myotubes; however, the compound was found to inhibit the differentiation of adipocytes in the 3T3-L1 cellular model. In high-fat-diet obese mice, treatment of DMC could improve glucose tolerance, reduce average weight, and increase the oxidation of fatty acids in muscle tissues. The mechanism of action was determined via FAO stimulation, which was mediated by AMPK activation in muscle [51].

In summary, there have been a large number of pieces of evidence about the antidiabetic and antiobesity properties of *S. nervosum* extract and its major constituent (DMC) in both *in vitro* and *in vivo* experimental setups. *S. nervosum* extracts were shown to inhibit polysaccharide hydrolytic enzymes, reduce blood sugar level, and protect pancreatic islets in rat models. On the other hand, the major chalcone DMC was capable of stimulating insulin secretion, increasing glucose uptake in differentiated adipocytes, and protecting pancreatic islet cells from oxidative stress, cellular damage, and glucotoxicity. Furthermore, DMC was also found to reduce lipid accumulation and promote fatty acid oxidation in the mouse. Although antidiabetic and antiobesity activities are not therapeutic indications of the plant in traditional medicine, these study results strongly suggested the potential clinical application of this plant in the fight against chronic conditions, such as diabetes and obesity. Clinical studies are required to confirm the pharmacological properties in humans.

### 6.3. Antioxidant Activities

Since the major components of *S. nervosum* are flavonoids, the extract and its constituents were also examined for antioxidant activities via different bioassays. The fruit extract of *S. nervosum* was shown to protect the kidney from cadmium-induced oxidative injury in rats. The oral administration of the extract (0.5–2.0 g/kg) clearly reduced the levels of blood urea nitrogen and plasma creatinine, which resulted in the restoration of creatinine clearance, in comparison with the cadmium alone-treated group. Histopathological examination also evidenced the protective effects of *S. nervosum* fruit extract (1-2 g/kg) against cadmium intoxication, specifically the diminishment of severe tubular necrosis, vacuolar degeneration, and glomerular structural changes. Further molecular investigation indicated that the fruit extract of *S. nervosum* reduced the levels of nitric oxide (NO) and malondialdehyde (MDA) and restored the levels of antioxidative enzymes, such as superoxide dismutase (SOD) and catalase (CAT), and nonenzymatic antioxidants (total, free, and protein thiols) [33].

The major compound, DMC (**38**), was also studied for the protective effects against H_2_O_2_-induced injuries in endothelial cells and hepatocytes. DMC showed potent ABTS radical scavenging activity (176.5 ± 5.2 *μ*mol Trolox equivalents/500 *μ*mol DMC), ferric reducing activity (213.3 ± 5.8 *μ*mol Trolox equivalents/500 *μ*mol DMC), and hydroxyl radical scavenging activity (IC_50_ 243.7 ± 6.3 *μ*M) in respective experiments. In the cellular assay, DMC was shown to protect human umbilical vein endothelial ECV-304 cells against H_2_O_2_-induced cytotoxicity through mechanisms of reducing intra- and extracellular ROS levels [52]. Pretreatment of DMC also demonstrated protective effects against H_2_O_2_-induced hepatotoxicity. In specific, DMC reduced the levels of NO and lactate dehydrogenase (LDH), inhibited the accumulation of MDA, and restored the reduced glutathione (GSH) level inside the liver cells [49].

As for other constituents, a study conducted by Min et al. [13] indicated that compounds **36** and **52** exhibited potent DPPH (1,1-diphenyl-2-picrylhydrazyl) radical scavenging activity with IC_50_ values of 22.8 and 27.1 *μ*M, respectively, which were comparable to that of positive control, *α*-tocopherol (20.1 *μ*M) [13]. Other flavonoids (**35** and **54**) also showed moderate antioxidant effects in different bioassays, including DPPH radical scavenging assay (IC_50_ 50.2 ± 2.8 *μ*M and 75.8 ± 2.5 *μ*M, respectively), superoxide radical scavenging assay (IC_50_ 56.3 ± 2.3 *μ*M and 317.5 ± 2.9 *μ*M, respectively), and lipid peroxidation (64.3 ± 2.5% and 60.3 ± 2.3% inhibition at 500 *μ*M, respectively) [36].

With the very high content of phenolic compounds, *S. nervosum* extract has shown potent antioxidant effects in various bioassays. The fruit extract showed its renal protection from cadmium intoxication in the rat model while DMC was found to protect endothelial cells and hepatocytes from H_2_O_2_-induced injuries. Since antioxidant activity is related to a wide range of pharmacological activities, such as anticancer, anti-inflammatory, hepatoprotective, and neuroprotective activities, the potent antioxidant activity may provide research ideas and directions for exploring other pharmacological effects of the plant and its constituents.

### 6.4. Neuroprotective Activities

The berry extract of *S. nervosum* was studied for its neuroprotective activities in the *Caenorhabditis elegans* model. As a result, the fruit extract was shown to prolong the average and maximum lifespan (test group: 27–30 days; control group: up to 22 days), as well as healthspan of the nematode by reducing the level of lipofuscin, which is an indicator of aging, and modulating the expression of healthspan-related genes. The detailed mechanism was determined to be DAF-16-interdependent by the upregulation of the family of transcription factors and the ability to extend the lifespan of DAF-16 mutant species. Furthermore, the extract was also capable of reducing the ROS level inside the nematode, which includes the involvement of skn-1 and sir-2.1 gene expression [53]. The fruit extract was also shown to inhibit glutamate-induced apoptosis by suppressing the endoplasmic reticulum stress, reduce the intracellular ROS level, and increase the expression of endogenous antioxidant enzymes, such as superoxide dismutase (SOD), catalase (CAT), and glutathione peroxidase (GPx), which was tested on mouse hippocampal neuronal cells (HT22) [34]. It can be seen that despite the limited number of publications on the neuroprotective effects of S. nervosum fruit extract, the initial data on the *C. elegans* model were shown very promising. Hence, the research area of the neuroprotective potential of *S. nervosum* should receive more attention from the scientific community. Extensive bioguided phytochemical studies should be carried out to discover the correlation of bioactive compounds and their respective mode of action.

### 6.5. Antiviral Activities

The antiviral properties of *S. nervosum* and its phenolic components were also investigated. Oh's group conducted bioactivity-directed isolation to identify the responsible compounds from *S. nervosum* flower buds by H1N1 neuraminidase inhibition assay. The most active compounds in H1N1 neuraminidase (wild type) assay were identified as C-methylated flavonoids with IC_50_ values ranging from 8.15 ± 1.05 to 93.77 ± 5.35 *μ*M. Interestingly, when tested on H274Y-mutant type (oseltamivir-resistant type), these compounds exhibited 2.46–7.00 times more potent, which was opposite to the oseltamivir case. Kinetic studies also revealed the underlying mechanism of these compounds as noncompetitive inhibitory mode [8]. The constituents from *S. nervosum* leaves were also found to inhibit the enzymatic activity of influenza neuraminidase. Noticeably, besides the C-methylated flavonoids, glycosides (**45** and **47**) were also active against neuraminidase [11]. A phloroglucinol-focused fractionation with the direction of QTOF/MS on *S. nervosum* leaves led to the isolation of four novel phloroglucinol compounds (**82**–**85**). Among them, cleistoperlone A (**82**) exhibited the strongest inhibitory effects on HSV-1 (herpes simplex virus type-1) in a cytopathic effect (CPE) reduction assay with the IC_50_ value of 7.50 ± 1.25 *μ*M and SI >13.33 [10].

One of the most common uses of *S. nervosum* in traditional Vietnamese and Chinese medicine is treating influenza. Current studies revealed that the C-methylated flavonoids were responsible compounds for anti-influenza activity of the extract. These findings may support the traditional claim of *S. nervosum* as an anti-influenza herb. However, published studies only reported the pharmacological results in enzymatic and cellular bioassays. Therefore, further *in vivo* studies should be conducted to confirm the anti-influenza effects of the plant in animal models, thereby laying the foundation for clinical trials.

### 6.6. Anti-Inflammatory Activities

The major component, DMC (**38**), was studied for its anti-inflammatory activities in multiple pathways. DMC was found to downregulate the expression of inflammatory mediators, including TNF-*α* (tumor necrosis factor-*α*), IL-1*β* (interleukine-1*β*), IL-6 (interleukine-6), and HMGB1 (High mobility group box 1), and reduce their production time- and dose-dependently in LPS-induced RAW264.7 macrophages. The detailed mechanistic study indicated that DMC suppressed the secretion of HMGB1, blocked the translocation of nucleocytoplasmic HMGB1, and interfered with the PI3K-PDK1-PKC*α* signaling pathway [54]. DMC also exhibited similar anti-inflammatory activities in LPS-stimulated rat Kupffer cells [55]. *S. nervosum* extract and DMC were also shown to attenuate LPS-stimulated inflammatory responses by inhibiting the production of inflammatory and proinflammatory mediators and activating the Nrf2/HO-1 pathway in macrophages. *In vivo* experiments showed that the oral administration of *S. nervosum* extract (100–200 mg/kg) reduced the mortality rate of experimental septic mice (60–80% survival rate), compared to 80% mortality rate in the control group. Histopathological examination also revealed the extract (200 mg/kg) ameliorated the swelling of the alveolar wall, alveolar congestion, and hepatic tissue damage in the experimental mice [12].

Previous studies have indicated the potential anti-inflammatory effects of *S. nervosum* extracts and DMC, which verified the traditional uses of the plant in folklore medicine, for example, skin diseases, pimples, and breast inflammation. *S. nervosum* extracts and its major flavonoid (DMC) were found to downregulate the expression of inflammatory and proinflammatory mediators. However, there have been limited animal studies on anti-inflammatory activities of the plant and isolated bioactive compounds.

### 6.7. Other Bioactivities

The aqueous extract of *S. nervosum* was found to improve the contraction of isolated rat heart, specifically increasing the contractility and reducing the contractile frequency pattern. A follow-up molecular experiment demonstrated that *S. nervosum* water extract inhibited Na^+^/K^+^-ATPase pump activities in a dose-dependent manner, which can explain the observed positive inotropic effects of the extract. The negative chronotropic effects were determined to relate with Ca^2+^-dependent ATPases, which eventually decreases the heart rate. The study suggested that the underlying mechanism of cardiotonic action of *S. nervosum* is more complex than that of ouabain, a well-known cardiac glycoside [29].

The antimicrobial activities of *S. nervosum* were also investigated. The methanol leave extract was tested against six strains of Gram-positive bacteria, two strains of Gram-negative bacteria, and one fungal species. Consequently, the extract (0.1 mg/mL) exhibited inhibitory activities against seven out of nine experimental bacterial species. Furthermore, the extract also showed anticaries activity against *Streptococcus mutans* (dental plaque organism) by inhibiting acid production and biofilm formation in a dose-dependent manner [56].

Tran et al. [24] published a study on antiosteoclastogenic activities of the ethanol extract of *S. nervosum* flower bud and its constituents. The extract was shown to suppress RANKL (receptor activator of nuclear factor-kappa-Β ligand)-induced osteoclast formation via inhibiting the activation of c-Fos (a proto-oncogene) and NFATc1 (nuclear factor of activated T cell cytoplasmic 1). The authors also conducted bioactivity-directed isolation to identify the active compounds for antiosteoclastogenic effects from *S. nervosum* extract. As a result, maslinic acid (**27**) and its two coumaroyl derivatives (**28** and **29**) were the responsible compounds [24].

The major chalcone-type compound from *S. nervosum*, DMC (**38**), was also studied for its cellular protective effects against H_2_O_2_-mediated cellular damage in pheochromocytoma cells (PC12). DMC increased the levels of glutathione and superoxide dismutase, leading to the reduction of endogenous ROS, which eventually protected cells from oxidative damage. Furthermore, DMC was also found to preserve mitochondrial membrane potential, which is an important indicator of cellular apoptotic activation, and decrease the caspase-3 activity in H_2_O_2_-treated PC12 cells [57].

Besides well-studied pharmacological effects like anticancer, antidiabetic, antioxidant, and anti-inflammatory activities, *S. nervosum* extract and its chemical compositions were also investigated in other bioassays, such as cardiotonic, antibacterial, anticaries, cellular protective, and antiosteoclastogenic activities. However, these studies are currently in the early stage, which requires further works on the mechanism of action and animal models to support future clinical application.

## 7. Conclusion and Perspective

In a nearly 20-year period, there have been a large number of studies conducted on *S. nervosum*, mainly in leaves and flower buds part. Phytochemical investigations have revealed that triterpenoids, flavonoids, and phloroglucinols are the main components of *S. nervosum*. Among them, C-methylated flavonoids stand out as bioactive compounds which are responsible for pharmacological activities of the plant, especially for the case of 2′,4′-dihydroxy-6′-methoxy-3′,5′-dimethylchalcone (DMC), while other major compound groups like triterpenoid and phloroglucinol were not paid enough attention in terms of pharmacological properties. Analytical studies reported the inconsistent content of DMC in *S. nervosum* flower buds, which suggested that DMC content might depend on plant varieties, location, and most importantly harvest time because the flavonoid content in flower buds can be changed in different flowering stages. Therefore, it is necessary to propose the standardization of varieties, geographical regions, and time of harvest. Such efforts will lay a practical basis for the development, modernization, and utilization of this plant in clinical practice.

*S. nervosum* extract and its major compound, DMC, were also investigated for pharmacological properties in various bioassays. The most noticeable bioactivities that should be mentioned are anticancer, antiviral, and antidiabetic activities. DMC showed potent cytotoxicity against various cancer cell lines via different mechanisms of action. In addition, DMC was found to reverse the drug-resistant abilities of cancer cells and significantly improve the anticancer effects of well-known drugs, such as 5-FU, doxorubicin, and Taxol. Some research groups successfully exploited the simplicity of the C-methylated chalcone backbone to synthesize analogues for significantly improved anticancer activities. This approach can be considered a promising direction for novel anticancer agent discovery.

Antiviral activities of *S. nervosum* also showed very promising results in *in vitro* setups, especially against influenza viruses (H1N1 and H9N2), which supported the traditional use of the plant against influenza. However, further animal and clinical studies are required before any clinical recommendation is made. Besides that, further pharmacological studies should focus on bioactivities of triterpenoids, phloroglucinols, and other flavonoid derivatives, which are also the major constituents of *S. nervosum* with undiscovered bioactive potentials.

As for the antidiabetic effect, *S. nervosum* and DMC were found to exhibit very promising activities in both *in vitro* and *in vivo* experiments. Although antidiabetes is not a curative indication of *S. nervosum* in traditional medicine, the pharmacological activity deserves more attention to identify the optimal dose and long-term effects in both animal and clinical studies. Further research should be performed to shed light on the clinical application of *S. nervosum* in the treatment of diabetes.

Although a large number of pharmacological studies were conducted, comprehensive studies to evaluate toxicity and safety of the plant are still absent. Long-term and short-term toxicity assessment should be carried out to identify potential risks and adverse effects of the plant and its constituents. Pharmacokinetic and pharmacodynamic studies are also very important to evaluate absorption, distribution, metabolism, and excretion of active substances, thereby providing dose estimation for humans.

In summary, the review provides an overview of ethnopharmacology and the current status of research on *S. nervosum*, including phytochemical, analytical, and pharmacological studies, which revealed the high potential of this medicinal plant for treatment of various diseases and conditions.

## Figures and Tables

**Table 1:** Chemical constituents from *S. nervosum*.

No.	Compounds	Parts	References
**1**	Cleistocalyxin	Leaves	[22]
**2**	Cleistocalyxic acid A	Leaves	[7]
**3**	Cleistocalyxic acid B	Leaves	[7]
**4**	3*β*-Hydroxytaraxast-20-en-28-oic acid	Leaves	[7]
**5**	3*β*,21*α*-Dihydroxytaraxast-28,20*β*-olide	Leaves	[7]
**6**	Cleistocalyxic acid C	Leaves	[7]
**7**	Cleistocalyxic acid D	Leaves	[7]
**8**	Cleistocalyxolide B	Leaves	[7]
**9**	11*α*,12*α*-Epoxy-2*α*,3*β*,23-trihydroxyolean-28,13*β*-olide	Leaves	[7]
**10**	Cleistocalyxic acid H	Leaves	[7]
**11**	2*α*-Hydroxymicromeric acid	Leaves	[7]
**12**	Actinidic acid	Leaves	[7]
**13**	Cleistocalyxic acid I	Leaves	[7]
**14**	Cleistocalyxic acid J	Leaves	[7]
**15**	Cleistocalyxic acid K	Leaves	[7]
**16**	Ursolic acid	Leaves, buds	[7, 23]
**17**	Corosolic acid	Leaves	[7]
**18**	Asiatic acid	Leaves	[7]
**19**	2*α*,3*β*,11*α*,23-Tetrahydroxyurs-12-en-28-oic acid	Leaves	[7]
**20**	2*α*,3*β*-Dihydroxy-24-nor-urs-4(23),12-dien-28-oic acid	Leaves	[7]
**21**	3*β*-Hydroxy-20-oxo-21(20⟶19)-abeo-taraxast-28,21*β*-olide	Leaves	[7]
**22**	*β*-Sitosterol	Leaves, buds	[22, 23]
**23**	Botulin	Leaves	[22]
**24**	Betulinic acid	Leaves	[7, 22]
**25**	Alphitolic acid	Leaves	[7]
**26**	Oleanolic acid	Leaves, buds	[7, 22, 24]
**27**	Maslinic acid	Leaves, buds	[7, 22, 24]
**28**	3-*O*-*trans*-*p*-Coumaroyl maslinic acid	Buds	[24]
**29**	3-*O*-*cis*-*p*-Coumaroyl maslinic acid	Buds	[24]
**30**	Arjunolic acid	Leaves	[7, 22]
**31**	Cleistocalyxic acid E	Leaves	[7]
**32**	Cleistocalyxic acid F	Leaves	[7]
**33**	Cleistocalyxic acid G	Leaves	[7]
**34**	2′,4′-Dihydroxy-6′-methoxy-3′,5′-dimethylchalcone	Leaves, buds, seed	[22, 23, 25]
**35**	3′-Formyl-4′,6′-dihydroxy-2′-methoxy-5′-methylchalcone	Buds	[26]
**36**	3′-Formyl-4′,6′,4-trihydroxy-2′-methoxy-5′-methylchalcone	Buds	[13]
**37**	3′-Formyl-6′,4-dihydroxy-2′-methoxy-5′-methylchalcone 4′-*O*-*β*-D-glucopyranoside	Buds	[13]
**38**	2′,4′-Dihydroxy-6′-methoxy-3′,5′-dimethylchalcone	Buds, leaves	[8, 11, 24]
**39**	2′,4′-Dihydroxy-3′-methyl-6′-methoxychalcone	Buds	[8]
**40**	(*E*)-4,2′,4′-Trihydroxy-6′-methoxy-3′,5′-dimethylchalcone	Buds	[8]
**41**	2,2′,4′-Trihydroxy-6′-methoxy-3′,5′-dimethylchalcone	Buds	[8]
**42**	Kaempferol	Leaves, buds	[22, 24, 26, 27]
**43**	Quercetin	Leaves, buds	[22, 24, 26, 27]
**44**	Tamarixetin	Buds	[26, 27]
**45**	Myricetin-3′-methylether 3-*O*-*β*-D-galactopyranoside	Leaves buds	[11, 26, 27]
**46**	Myricetin-3′-methylether	Buds	[26]
**47**	Myricetin-3′,5′-dimethylether 3-*O*-*β*-D-galactopyranoside	Leaves, buds	[11, 27]
**48**	5,7,8,4′-Tetrahydroxy-3′,5′-dimethoxyflavone-3-*O*-*β*-D-galactopyranoside	Buds	[26]
**49**	Gossypetin-8,3′-dimethylether-3-*O*-*β*-D-galactoside	Buds	[26]
**50**	Luteolin	Buds	[24]
**51**	7-Hydroxy-5-methoxy-6,8-dimethylflavone	Buds	[8, 26]
**52**	(2*S*)-8-Formyl-6-methylnaringenin	Buds	[13]
**53**	(2*S*)-8-Formyl-6-methylnaringenin 7-*O*-*β*-D-glucopyranoside	Buds	[13]
**54**	(2*S*)-8-Formyl-5-hydroxy-7-methoxy-6-methylflavanone	Buds	[8, 26]
**55**	8-Formyl-5,7-dihydroxyl-6-methylflavanone	Buds	[26]
**56**	(2*S*)-6-Formyl-8-methyl-7-*O*-methylpinocembrin	Buds	[8]
**57**	(2*S*)-7,2′-Dihydroxy-5-methoxy-6,8-dimethylflavanone	Leaves	[11]
**58**	(2*S*)-7-Hydroxy-5-methoxy-6,8-dimethylflavanone	Leaves	[11]
**59**	(2*S*)-7-Hydroxy-5-methoxy-8-methylflavanone	Buds	[8]
**60**	(2*S*)-8-methylpinocembrin	Buds	[8]
**61**	(2*S*)-5-Hydroxy-7-methoxy-6,8-dimethylflavanone	Buds	[8]
**62**	(2*S*)-5,7-Dihydroxy-6,8-dimethylflavanone	Buds	[8]
**63**	(2*S*)-2,7-Dihydroxy-5-methoxy-6,8-dimethylflavanone	Buds	[8]
**64**	(2*S*,3*S*)-2,3-*trans*-5,7-Dihydroxy-6,8-dimethyldihydroflavonol	Buds	[8]
**65**	7-Hydroxy-5-methoxy-6,8-dimethylisoflavone	Leaves, buds	[8, 11]
**66**	Hariganetin	Seed	[25]
**67**	3-*O*-Methylellagic acid-4′-*O*-*α*-ʟ-rhamnopyranoside	Leaves	[11]
**68**	(2*S*)-2,6-Dihydroxy-4-methoxy-5,7-dimethylcoumaran-3-one	Buds, leaves	[11, 24]
**69**	2,4′,6′-Trihydroxy-2′-methoxy-3′,5′-dimethylacetophenone	Leaves	[11]
**70**	1-[(2-Methylbutyryl)-phloroglucinyl]-*β*-D-glucopyranoside	Buds	[24]
**71**	(*trans*)-4-Hydroxy-5-(2-hydroxypropan-2-yl)-2-methylcyclopent-2-en-1-one	Buds	[24]
**72**	4-Hydroxy-2,2,5-trimethylcyclopent-4-ene-1,3-dione	Buds	[24]
**73**	4′-Dihydrophaseic acid	Buds	[24]
**74**	5-(3*S*,8*S*-Dihydroxy-1*R*,5*S*-dimethyl-7-oxa-6-oxobicyclo [1–3]-oct-8-yl)-3-methyl-2*Z*,4*E-*Pentadienoic acid	Buds	[24]
**75**	4-Hydroxybenzoic acid	Buds	[24]
**76**	Methyl 3,4,5-trihydroxybenzoate	Buds	[24]
**77**	4-Hydroxy-3,5-dimethoxybenzoic acid	Buds	[24]
**78**	3-Hydroxy-3-phenylpropanoic acid	Buds	[24]
**79**	Cinnamic acid	Buds	[24]
**80**	Cleistocaltone A	Buds	[9]
**81**	Cleistocaltone B	Buds	[9]
**82**	Cleistoperlone A	Leaves	[10]
**83**	Cleistoperlone B	Leaves	[10]
**84**	Operculatol A	Leaves	[10]
**85**	Operculatol B	Leaves	[10]
**86**	1-Tetratriacontanol	Leaves	[22]

**Table 2:** Cytotoxicity (IC_50_) of DMC against different cancer cell lines.

Cancer cell line	Description	IC_50_ (*μ*m)	Reference
P-388	Mouse lymphoid neoplasm	10.31	[25]
KB	Human epidermoid carcinoma in the mouth	15.90	—
MCF-7	Human breast cancer	14.51	—
A549	Human lung cancer	13.04	—
ASK	Rat glioma	9.00	—
PANC-1	Human pancreas	10.5 ± 0.8	[39]
MIA-PACA2	Human pancreatic carcinoma	12.2 ± 0.9	—
SMMC-7721	Liver cancer	32.3 ± 1.13	[23]
8898	Pancreas cancer	37.2 ± 2.15	—
SPC-A-1	Lung cancer	84.6 ± 4.36	—
HeLa	Tumor of cervix uteri	37.7 ± 1.64	—
95-D	High metastatic lung carcinoma	84.8 ± 4.71	—
GBC-SD	Gall bladder carcinoma	81.3 ± 2.75	—
A549	Human lung cancer	2.3 ± 0.44	[38]
HepG2	Human liver cancer	8.3 ± 0.22	—
MCF-7	Human breast cancer	15.1 ± 0.72	—
HeLa	Tumor of cervix uteri	11.8 ± 0.42	—
HeLa/Tax	Taxol-resistant HeLa cells	15.3 ± 0.19	—

## Abbreviations

5-FU: Fluorouracil
DMC: 2′,4′-Dihydroxy-6′-methoxy-3′,5′-dimethylchalcone
MS: Mass spectrometry
NMR: Nuclear magnetic resonance
RANKL: Receptor activator of nuclear factor-kappa-Β ligand
ROS: Reactive oxygen species
STZ: Streptozotocin.
